# Brain signature of sleep quality

**DOI:** 10.1093/sleep/zsaf200

**Published:** 2025-07-22

**Authors:** Wen Liu, Vincent Küppers, Masoud Tahmasian

**Affiliations:** Institute of Neuroscience and Medicine, Brain and Behaviour (INM-7), Research Center Jülich, Jülich, Germany; Institute of Systems Neuroscience, Medical Faculty and University Hospital Düsseldorf, Heinrich Heine University, Düsseldorf, Germany; Institute of Neuroscience and Medicine, Brain and Behaviour (INM-7), Research Center Jülich, Jülich, Germany; Institute of Systems Neuroscience, Medical Faculty and University Hospital Düsseldorf, Heinrich Heine University, Düsseldorf, Germany; Department of Nuclear Medicine, University Hospital and Medical Faculty, University of Cologne, Cologne, Germany; Institute of Neuroscience and Medicine, Brain and Behaviour (INM-7), Research Center Jülich, Jülich, Germany; Institute of Systems Neuroscience, Medical Faculty and University Hospital Düsseldorf, Heinrich Heine University, Düsseldorf, Germany; Department of Nuclear Medicine, University Hospital and Medical Faculty, University of Cologne, Cologne, Germany

Healthy sleep is a fundamental pillar of various neurophysiological, emotional, and cognitive processes, including emotion regulation, memory consolidation, and brain waste clearance [1–3]. Sleep quality is a multidimensional concept that has been widely assessed using the Pittsburgh Sleep Quality Index (PSQI) [4, 5]. It encompasses seven components of self-reported sleep quality, sleep latency, sleep duration, habitual sleep efficiency, sleep disturbances, use of sleeping medication, and daytime dysfunction [5]. However, the neurobiological substrates of sleep quality remain elusive.

Recently, Monti employed a data-driven approach to investigate the association between brain shape and sleep quality in healthy individuals from the Human Connectome Project Young Adults (HCP-YA) dataset (*n* = 1112) [6]. Using principal component analysis (PCA), Monti identified two principal components (PCs). PC1 primarily reflected self-reported sleep quality, sleep duration, and habitual sleep efficiency, while PC2 encompassed sleep onset latency, sleep disturbances, and use of sleep medications. Interestingly, both PSQI total score and PC1 were negatively associated with the shape of the basal ganglia, particularly the bilateral putamen and caudate. PC1 showed a negative correlation with the left pallidum, but no significant associations were observed between either PSQI total score or PC1 and the shape of cortical regions, or between PC2 and the shape of either cortical or subcortical structures. Although inconsistencies across studies underscore the complex nature of sleep–brain associations [7–9], this study provides novel insights into the intricate relationship between particular sleep quality components and subcortical brain structures.

The basal ganglia and subthalamic nucleus are involved in sleep regulation through their role in neural oscillations within the thalamo-cortical-basal ganglia network [10]. Genetic studies have demonstrated the enrichment of insomnia-associated genes in both cortical and basal ganglia structures, particularly within the striatum, comprising the caudate and putamen [11]. A task-fMRI study also suggested attenuated recruitment of the caudate as a potential trait marker for developing insomnia disorder [12]. Furthermore, the left sensorimotor globus pallidus has even been suggested as a target for deep brain stimulation to improve sleep quality in patients with Parkinson’s disease after a 2-year follow-up [13]. Together, these findings underscore the crucial involvement of the basal ganglia in poor sleep quality and insomnia. However, the neural correlates of sleep quality extend beyond these regions. Volumetric reductions in the hippocampus, amygdala, and thalamus have been reported in chronic insomnia disorder as well [14]. Using different morphological metrics from Monti’s, studies with community-based samples have identified negative associations between sleep quality and both cortical volume and surface area in the right temporal lobe [15], as well as associations with reduced right superior frontal volume and increased atrophy rate in widespread frontal, temporal, and parietal lobes [9]. In addition to using different morphological metrics, another potential contributor to inconsistent neuroimaging findings is the multifaceted nature of sleep quality, with neural correlates varying by specific sleep characteristics. For example, short sleep duration (<7 h) is linked to reduced gray matter volume in temporal and occipital cortices, while long sleep duration (>9 h) has been associated with increased volume in the basal ganglia [8]. Furthermore, subcortical volumes in the amygdala, hippocampus, caudate, and thalamus are linked with sleep duration only among long sleepers (≥7 h) [16]. Distinct neuroanatomical patterns have been observed among subtypes of insomnia disorder [17]. Specifically, paradoxical insomnia (i.e., misperception of sleep quality) is associated with morphological alterations in the caudate, putamen, and nucleus accumbens, while psychophysiological insomnia, characterized by heightened anxiety related to the sleep time and bedroom environment, is linked to shape abnormalities in the thalamus, amygdala, and hippocampus [17]. Although cortical thinning in the anterior cingulate cortex and inward shape deformation in the head of the right caudate have been observed among insomnia patients, these alterations were not significantly associated with sleep quality [18]. A recent large-scale meta-analysis identified convergent alterations in the bilateral subgenual anterior cingulate cortex, right amygdala, and hippocampus across different sleep disorders [19]. However, some large-scale studies identified no linear association between insomnia and brain structures [8, 20], highlighting the need for multivariate and nonlinear analyses to better capture the complex sleep–brain interactions. Other contributing factors to such inconsistency may include the scale to assess sleep quality, the characteristics of the sample population (e.g., healthy individuals, the general population, or clinical patients), as well as variability in preprocessing and neuroimaging analysis methods [19].

To address high intercorrelations among PSQI’s seven components, Monti employed PCA with varimax rotation to reduce dimensionality and improve interpretability, while capturing the multidimensional nature of sleep quality [6]. Alternatively, Cole et al. applied exploratory factor analysis to the PSQI and identified a different component structure, grouping self-reported sleep quality with sleep latency [21]. The differences may reflect age-related variations in sleep patterns and the methodological distinctions. Participants in Monti’s study were 22–35, whereas Cole’s participants were over 60 years old. Sleep patterns vary across the lifespan, as shown in a study of individuals aged 18–98 that identified four PSQI-based sleep profiles [22]. Delayed sleepers—characterized by poor sleep quality and prolonged latency—were more common in younger adults (<62 years), while poor sleepers were more prevalent among older adults (>62 years) [22]. Beyond the demographic differences, methodological choices may also account for the divergent findings. PCA captures both shared and unique variance for dimensionality reduction, while factor analysis focuses only on the shared variance to identify underlying latent constructs [23]. Of note, neither approach is inherently superior; the choice depends on the study’s objectives.

Several potential directions may be worth considering in the future. First, to better capture the multidimensional nature of sleep quality and complex latent sleep–brain interactions, further studies would benefit from integrating a multimodal neuroimaging approach and adopting stratified, multivariate analytical frameworks, particularly those incorporating dimensionality reduction while simultaneously extracting latent constructs, which may help uncover hidden sleep–brain associations. Since PCA yields components with both positive and negative loadings and does not guarantee coherent clusters, orthogonal nonnegative matrix factorization (OPNMF) offers a complementary approach [24]. By decomposing data into sparse, nonnegative, and orthogonal components, OPNMF can improve interpretability and generalizability [25]. Second, given the inconsistent findings regarding the neural substrates of sleep across small/medium-sized samples, large-scale neuroimaging meta-analysis, big data analysis, cross-validation, replication, and generalization (e.g., out-of-cohort validation) of observed findings are essential [19, 26–28]. These strategies enhance generalizability and reduce the risk of overfitting, thereby supporting the identification of robust and replicable neural correlates of sleep. Third, as sleep patterns vary drastically across different countries [29, 30], future studies should leverage multicenter, cross-cultural cohorts (e.g., the ENIGMA-Sleep working group [31] or other international initiatives). Fourth, given the multidimensionality of sleep quality and variability across dimensionality reduction methods, systematic comparisons of dimension reduction methods (e.g., PCA, factor analysis, or OPNMF) are needed, along with detailed explanations and interpretations of the resulting components. Fifth, as Monti’s study included only young healthy adults, expanding investigations to include broader age groups, such as children, adolescents, middle-age, and older adults from the general population, as well as clinical samples (e.g., chronic insomnia disorder), is necessary to capture age-specific effects and the role of neuropsychiatric comorbidities on sleep–brain interaction. Sixth, advanced machine learning and artificial intelligence approaches may help unravel the complex, nonlinear sleep–brain relationships at the individual person level, supporting efforts toward precision medicine insights [32]. Finally, longitudinal studies should assess the dynamic interplay between sleep health and brain structures over time.
